# Transcriptional alterations reveal Bacillus amyloliquefaciens-rice cooperation under salt stress

**DOI:** 10.1038/s41598-019-48309-8

**Published:** 2019-08-15

**Authors:** Puneet Singh Chauhan, Charu Lata, Shalini Tiwari, Abhishek Singh Chauhan, Shashank Kumar Mishra, Lalit Agrawal, Debasis Chakrabarty, Chandra Shekhar Nautiyal

**Affiliations:** 1 CSIR-National Botanical Research Institute, Rana Pratap Marg, Lucknow, 226001 India; 2 Academy of Scientific and Innovative Research (AcSIR), Sector 19, Kamla Nehru Nagar, Ghaziabad, Uttar Pradesh 201 002 India; 3 Present Address: Doon University, Mothorowala Road, Kedarpur, Uttarakhand 248001 India

**Keywords:** Plant signalling, Abiotic

## Abstract

The *Bacillus amyloliquefaciens*-SN13 and model crop rice (*Oryza sativa*) were chosen to understand the complex regulatory networks that govern plant-PGPR interaction under salt stress. During stress, inoculation with SN13 significantly increased biomass, relative water content, proline and total soluble sugar in rice while decreased lipid peroxidation and electrolyte leakage. Extensive alterations in gene expression were also observed in rice root transcriptome under stress in the presence of SN13. Rhizobacteria induced changes in expression of a considerable number of photosynthesis, hormone, and stress-responsive genes, in addition to cell-wall and lipid metabolism-related genes under salt stress as compared to salt stress or SN13 inoculation alone, indicating its potential role in reducing the harmful effects of salinity. To validate RNA-seq data, qRT-PCR was performed for selected differentially expressed genes representing various functional categories including metabolism, regulation, stress-response, and transporters. Results indicate qualitative and quantitative differences between roots responses to SN13 under stressed and unstressed conditions. Functional expressions of *OsNAM* and *OsGRAM* in yeast showed enhanced tolerance to various abiotic stresses, indicating crucial SN13-rice interaction in imparting beneficial effects under stress. This is first detailed report on understanding molecular mechanism underlying beneficial plant-microbe interaction in any economically important model crop plant under abiotic stress.

## Introduction

Salinity is one of the major stress factor that pose a serious threat to sustainable agricultural production and global food security by causing yield loss of cereal crops^1^. By the year 2050, more than 50% of the total cultivable lands are reported to face the serious threat of salinization^2^. Increasing soil salinization in irrigated as well as rainfed agriculture particularly in coastal areas has necessitated either the introduction of salt tolerant varieties or the use of improved management practices in the affected regions^3^. Development of varieties for improved salt tolerance through transgenic technology or conventional breeding requires identification of traits or genes that could confer resistance to salinity^1^. However, both these approaches are labour and cost intensive as well as time-consuming. On the other hand, the use of better irrigation management practices in salinity affected areas has been demonstrated to be mostly unfeasible on accounts of the cost involved as well as its implementation on a larger scale^3^. Considering the above factors, it has now become a prerequisite to use alternate technologies for sustained agricultural production such as the use of plant growth promoting rhizobacteria (PGPR) for salt stress amelioration. This technology also stands significant these days from the perspective of global climate change and disproportionate fertilizer use in agriculture^4^.

PGPR are usually rhizosphere colonizing bacteria that are thought to communicate with host plants via secreting signalling factors in the form of proteins, metabolites and/or volatile organic compounds (VOCs) and promote plant growth by conferring beneficial effects through various primary and secondary mechanisms such as siderophore and auxin production, biofilm formation, and exopolysaccharide secretion^4,5^. Several recent studies have reported PGPR to be likely elicitors for abiotic stress tolerance including salinity and drought^6,7^. However, plant-PGPR interaction in rhizosphere for abiotic stress tolerance is not an outcome of classic “gene-to-gene” interaction at the molecular level as abiotic stresses are usually quantitative in nature and adaptation to such stresses are usually a part of the multigenic response^6,8^. However, not much work has been carried out on elucidating the stress tolerance mechanisms initiated by various PGPR which are thought to be distinct owing to the diverse and evolutionary uniqueness of each bacterial genus. Therefore, elucidation of the complex gene regulatory network(s) governing rhizosphere signalling during plant-PGPR interaction(s) is a subject of intense research. Transcriptome analysis through high throughput sequencing and discovery of candidate genes involved in plant-PGPR interactions can thus help us to better understand the complex networks that regulate root-rhizosphere signalling. Recently transcriptome profiling of *Arabidopsis thaliana* in the presence of *Bacillus amyloliquefaciens* FZB42 has been reported under salt stress^9^. However, no such study has been carried out in any cereal crop so far. *Bacillus* sp. is one of the largest genus of Gram-positive PGPR with phytobeneficial traits that are known to naturally inhabit arable lands^4^. A *Bacillus amyloliquefaciens* strain NBRISN13 (SN13; GenBank Accession: KC293995) was characterized for various growth promotory attributes and salt stress tolerance in a previous study from our laboratory^4^.

Further, due to its significant phytobeneficial traits and abiotic stress tolerance characteristics, SN13 has not only emerged as an excellent PGPR for rice which is generally very sensitive to salt stress at young seedling stage but has also opened up the area of research pertaining to deciphering the complex molecular networks of perception, response, and adaptation to abiotic stress(s). Thus a comprehensive insight into the molecular mechanisms underlying PGPR-induced salt stress tolerance in rice will definitely facilitate crop improvement programs. In this study, the transcriptome response of roots of an Indian rice cultivar under salt stress in the presence or absence of SN13 was carried out on Illumina HiSeq. 2000 Platform. Further candidate stress-responsive genes with potential roles in controlling plant-PGPR interaction were selected, annotated and validated using qRT-PCR. Functional characterization of selected SN13-responsive genes namely *OsNAM* (No Apical Meristem) and *OsGRAM* (Glucosyltransferases, Rab-like GTPase activators, Myotubularin) was done by heterologous expression in yeast. Therefore, present study aimed towards understanding the detailed molecular mechanism underlying beneficial microbe-plant interaction in rice crop exposed to salt stress.

## Materials and Methods

### Plant material, growth conditions, and stress treatment

A widely cultivated rice cv. ‘Saryu 52’ was used to examine the transcriptome responses upon SN13 inoculation. The experiment was conducted with three replications in a growth chamber at 70% relative humidity and 260–350 µE m^2^ s^−1^ light intensity, of CSIR-NBRI Lucknow, India under standard conditions. The experiment was designed with four treatments namely, control, salt stress (100 mM NaCl), SN13 (1% inoculum) and salt + SN13 (100 mM NaCl and 1% inoculum). Sterilized seeds were allowed to germinate in the dark for three days, and sown to raise a nursery bed for one week after which the seedlings were transplanted to Hewitt medium and grown hydroponically. After 24 h, 1% SN13 suspension [~10^9^ CFUmL^−1^ (colony forming unit/mL); 28 °C] grown in nutrient broth, was inoculated to the trays designated for SN13 and salt + SN13 seedlings. After 24 h of inoculation, salt and salt + SN13 seedlings were supplied with 100 mM of salt (NaCl) for 7 days. To avoid any diurnal variation, all tissues were harvested at the same time, and biochemical assays were performed on the same day of harvesting. Root tissues for RNA sequencing and qRT-PCR analyses were frozen in liquid nitrogen and kept at −80 °C until further use.

### Phenotypic characterization of rice seedlings based on important abiotic stress markers

Relative water content (RWC) and electrolyte leakage (EL) were determined in leaf and root samples, respectively in all four treatments as described by Lata *et al*.^10^. The lipid peroxidation (LP) level was assessed by measuring malondialdehyde (MDA) content in control and stressed leaf samples^11^. Proline content and total soluble sugar (TSS) were determined as described elsewhere^6,12^. Chlorophyll content was evaluated according to Wellburn with some modifications^13^. All the absorbance was recorded in a microplate reader (Spectrum max plus; Molecular devices, California, US). All experimental data were the averages of three independent biological replicates expressed with standard deviation (average ± SD). One-way ANOVA was used to test the significance among samples. Duncan multiple range test at P < 0.05 was carried out with the help of SPSS software version 16.0 (SPSS Inc./IBM Corp. Chicago, USA) to compare among the means, and the Graph Pad Prism software (version 5.03, San Diego, USA) was used to graphically illustrate the results.

### RNA isolation, cDNA library preparation, and sequencing

Root samples were used for total RNA extraction using Spectrum^™^ Plant Total RNA Kit (Sigma, USA) which was then subjected to DNase treatment using TURBO DNase (Ambion, USA) for removal of DNA contamination. The integrity (RIN value: ≥8.0) and concentration of the RNA samples (0.2–1.5 µg/µl) were determined on an Agilent Bioanalyzer 2100 (Agilent Technologies) using the RNA 6000 Nano kit. Four separate RNA-Seq libraries were prepared using the True-Seq^TM^ RNA sample preparation kit (Illumina CA, USA) from the normalized starting quantities of the total RNA from each of the four samples. Three biological replicates of each sample were pooled together to constitute the final four samples for sequencing. Library preparations and sequencing were performed by Sandor Life Sciences Pvt. Ltd. on the HiSeq2000 sequencing system (Illumina) using Illumina TruSeq protocol and Illumina TruSeq RNA-Seq adapter with 100 bp paired-end reads. Raw data obtained from sequencing were converted to the Fastq format and deposited to the NCBI Sequencing Read Archive database (SRA: SRP089905, Bioproject: PRJNA343061).

### Analysis of high‐throughput data

The qualities of four libraries prepared were visualized using Fastq files, and raw reads were analyzed using NGSQCTOOLKIT (http://www.nipgr.res.in/ngsqctoolkit.html). The filtered reads were then mapped onto coding sequences of rice (*Oryza sativa* L.) downloaded from the Ensembl Plants (http://plants.ensembl.org/Oryza_sativa/Info/Index) using Bowtie and TopHat v2.0.8 with default settings^14^. Differential gene expression levels among the four libraries in the rice genome were determined using the Cuffdiff software within Cufflink 2.2.0 release^15^, and fragment per kilobase mapped (FPKM) values for each transcript were also calculated. Fold change values were calculated as the ratio of FPKM of different samples to that of control. Transcripts with ≥2.0-fold change values were considered up-regulated and those with ≤2.0 down-regulated. A gene was identified as significantly differentially expressed with a false discovery rate (FDR) of the Benjamini-Hochberg multiple tests of 5% (P < 0.05)^16^. PageMan (http://mapman.mpimp-golm.mpg.de/pageman/) was used to analyze the expression profiles of different clusters of differentially expressed genes (DEGs).

The gene ontology assignment was carried out following the Gene Ontology (GO) guidelines of the Blast2GO program^17^. The annotations of gene function(s) were also obtained from the Rice Genome Annotation Project database release 7 (http://rice.plantbiology.msu.edu/). Pathway assignments were performed following the Kyoto Encyclopedia of Genes and Genomes (KEGG) mapping (http://www.genome.ad.jp/kegg/kegg2.html)^18^. Metabolic and gene regulatory network(s) for rice, salt, and SN13 interactions were generated using the MapMan v 3.6.0RC1 using all DEGs identified through Cuffdiff program with ≥2.0-fold change^19^.

### Quantitative real-time (qRT) PCR analysis

One microgram of DNase treated total RNA was used for first strand cDNA synthesis using Maxima H Minus M-MuLV reverse transcriptase (Thermo Scientific, USA). A 5-fold dilution of cDNA samples was done with deionized water (Sigma, USA) to be used in qRT-PCR reactions. qRT-PCR was carried out using 2X Brilliant III SYBR® Green Q PCR (Agilent Technologies, USA) on a Stratagene Mx3000P (Agilent, USA) with cycling conditions and replications as described in Tiwari *et al*.^20^. The amount of transcript accumulated for each target gene normalized to the reference gene *Actin*. The qRT- PCR primers were designed using the IDT Primer Quest software with default parameters: Tm = 59–62 °C; GC = 45–55%; amplicon size = 100–150 bp (Table S1). The heatmap for expression profiles of DEGs was generated using the MEV4 software package^21^.

### Cloning full-length cDNAs of OsNAM and OsGRAM gene

Gene-specific primers including nested primers were designed manually to amplify and isolate gene encoding NAM (LOC_Os11g03370) and GRAM domain containing protein (LOC_Os03g08860). Full length of *OsNAM* and *OsGRAM* genes were amplified from rice roots, cloned in pGEM®-T Easy Vector (Promega), transformed into *E. coli* DH5α separately and sequenced using M13 universal primers. Further, the genes were cloned into the pYES2 vector using *BamHI* and *XbaI* restriction sites under the control of a T7 promoter to obtain *pYES2-OsNAM* and *pYES2-OsGRAM* constructs. The primers used for the cloning are listed in Table S1.

### Transformation of OsNAM and OsGRAM gene in Saccharomyces cerevisiae and stress tolerance assays

For functional characterization, both the genes were cloned into a pYES2 vector and transformed in yeast strain *S. cerevisiae* (INVSc1). INVSc1 transformed pYES2 vector (empty vector; EV) was used as a control. All the transformed cells were inoculated in Synthetic Complete (SC) medium containing 2% glucose at 30 °C overnight at 220 rpm. Late exponential phase cells were diluted at a concentration of 1 × 10^7^ cells/ml in the medium and further an equal volume of each suspension was inoculated in SC medium with 2% galactose with varying concentrations of glycerol, mannitol, NaCl, arsenate and arsenite viz 2, 4, 6, 8 and 10% of glycerol, 0.25, 0.5, 0.75 and 1 M of mannitol and 0.5, 1, 1.5, 2 and 2.5 M NaCl were incubated at 30 °C for 24 h at 220 rpm. Yeast cells carrying *pYES2*, *pYES2-OsNAM* and *pYES2-OsGRAM* were also grown at different temperatures ranging from 25 °C to 45 °C to monitor their heat tolerance capacity. The growth of all yeast cells was monitored spectrophotometrically at 600 nm after 24 h.

## Results and Discussion

### SN13 inoculation modulates morpho-physiological and biochemical parameters in rice during salt stress

**Figure 1:**
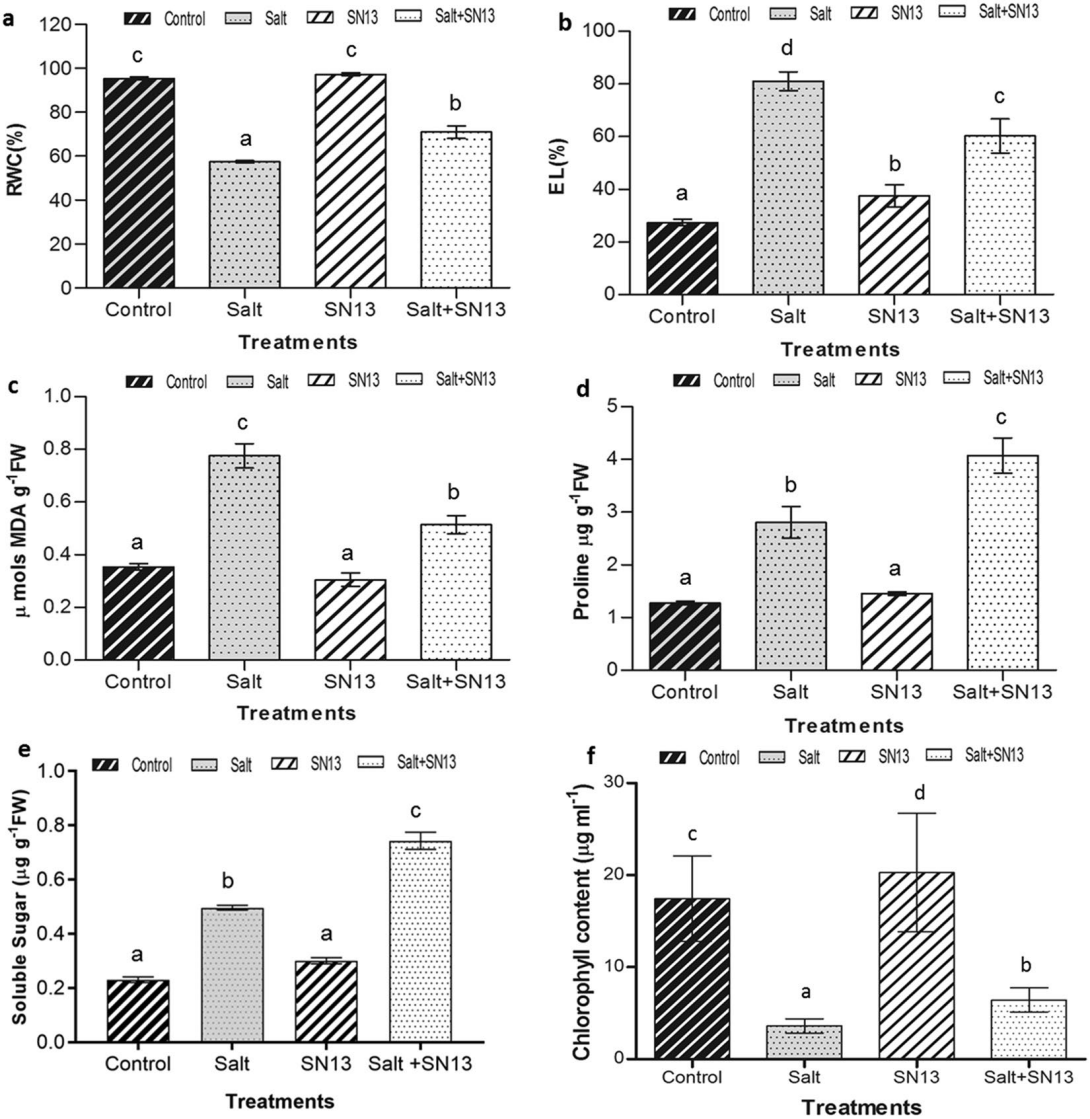
Determination of RWC (**a**), EL (**b)**, LP (**c**), Proline (**d**), Total soluble sugar (**e**) and chlorophyll (**f**) in cv. Saryu 52 exposed to 100 mM salt stress at 7 days in the presence and absence of SN13. Data represent the means ± SD of three independent experiments. Different letters on the graph indicate significant differences according to Duncan’s test (P ≤ 0.05).

One-week-old rice seedlings were subjected to 7 days of salt stress with and without SN13 inoculation to determine the effects of SN13**-**inoculation on root and shoot growth as well as biomass. Since rice seedlings at 7- to 14-day old stage are very delicate and sensitive to abiotic stresses as apparent from several previous studies^22,23^, this stage was chosen for our salt stress experiments. Compared to uninoculated seedlings, inoculated ones showed no statistical difference in shoot length and root length under both stressed and unstressed condition. However, ~48% and ~24% increase in fresh weight and dry weight were recorded in inoculated seedlings in comparison to uninoculated seedlings under both stressed and unstressed condition indicating SN13-inoculation improves stress endurance capacity of rice (Fig. S1 and Table S2). Increased biomass was also observed in inoculated chickpea and maize under drought and salt stress respectively^6,24^. Plant water status and membrane integrity of rice seedlings were determined through estimating RWC and EL. SN13-inoculated salt-stressed rice seedlings showed ~26% and ~23% lesser decline in RWC as compared to control and salt-stressed seedlings respectively, suggesting that SN13 inoculation improves and/or maintains plant water balance in rice under stress (Fig. 1a). Similarly, SN13-inoculated rice seedlings showed ~76% less electrolyte leakage and an approximately 34% reduction in the level of lipid peroxidation as compared to salt-stressed plants suggesting a significant role of SN13 in maintaining membrane integrity (Fig. 1b,c). Similarly, Kakar *et al*.^25^. observed a reduction in EL and MDA content on the application of PGPR under drought stress in rice. However, a slight increase in the level of EL in SN13 inoculated seedlings as compared to uninoculated seedlings under unstressed condition is indicative of bacterial adherence to root or root cortex^20,26^. Further, leaves of cv. Saryu 52 also showed significantly increased accumulation of proline upon salt stress with (219%) and without (120%) SN13-inoculation when compared to control suggesting that SN13 helps in ameliorating salt stress conditions in rice (Fig. 1d)^4,24^. Similarly, TSS content was also found to be elevated by 49% in inoculated seedlings than uninoculated seedlings under salt stress (Fig. 1e). In the presence of PGPR higher accumulation of TSS was also reported under salinity and oxidative stress by Kang *et al*.^27^. An estimation of the total chlorophyll content suggested an increase in the pigment by 44% in SN13-inoculated seedlings than the uninoculated seedlings under salt stress. Higher chlorophyll content in the SN13-inoculated seedlings could be due to the bacterial 1-aminocyclopropane-1-carboxylic acid deaminase (ACD) activity which has been associated with mitigating the effects of salt stress on chlorophyll (Fig. 1f)^24^.

Overall these morpho-physiological and biochemical results support the earlier known characteristics of SN13 in imparting beneficial effects on rice seedlings subjected to salt stress by maintaining water balance and protecting them from membrane damage as well as osmotic stress^4^.

### Transcriptome sequencing and mapping onto O. sativa reference genome

**Figure 2:**
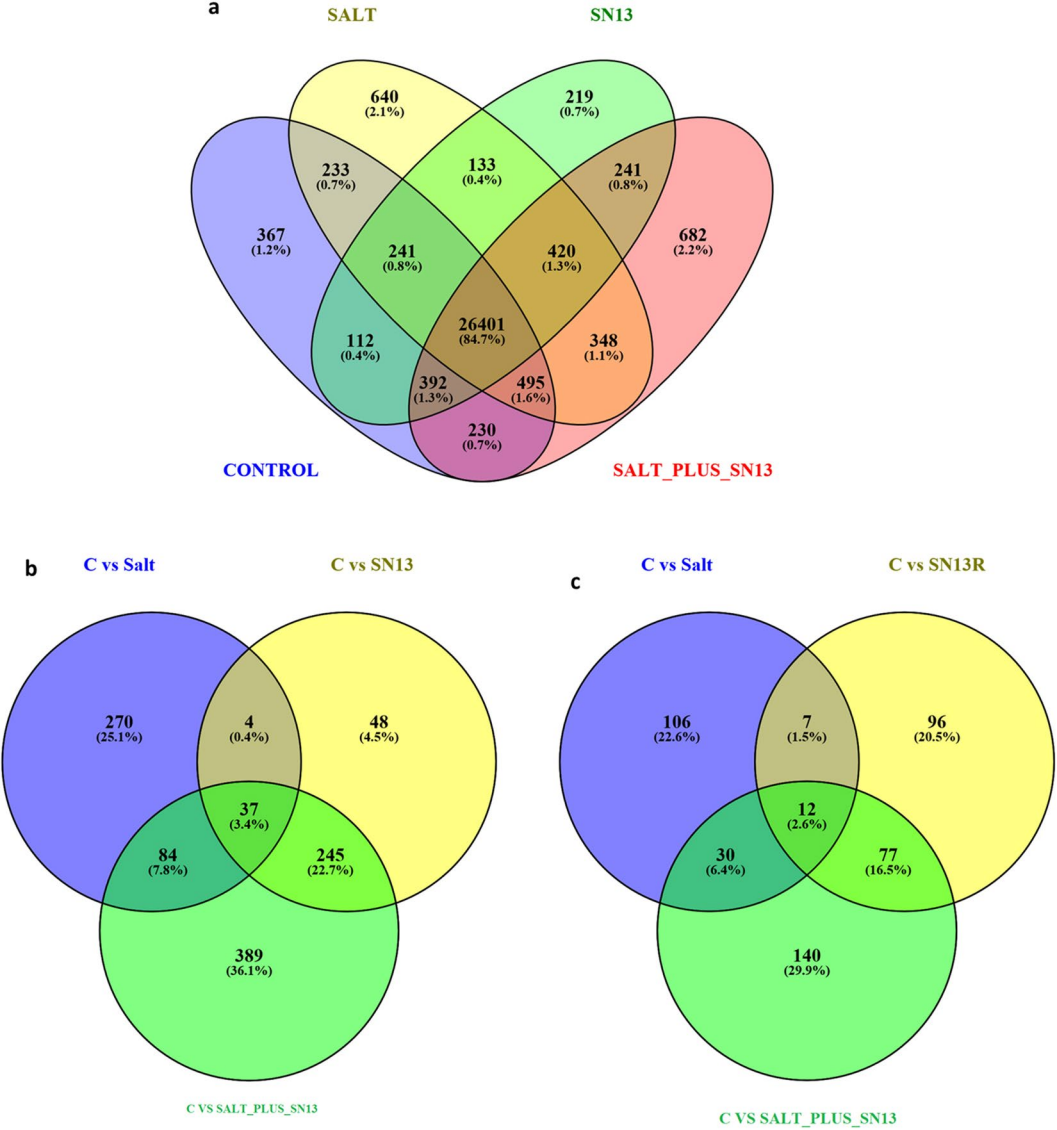
Venn diagram of the total number of expressed non-redundant transcripts in control, salt, SN13 and salt + SN13 samples (**a**). Venn diagram of the differential expressed up-regulated genes (**b**) and downregulated genes (**c**) in salt, SN13 and salt + SN13 samples as compared to control.

**Figure 3:**
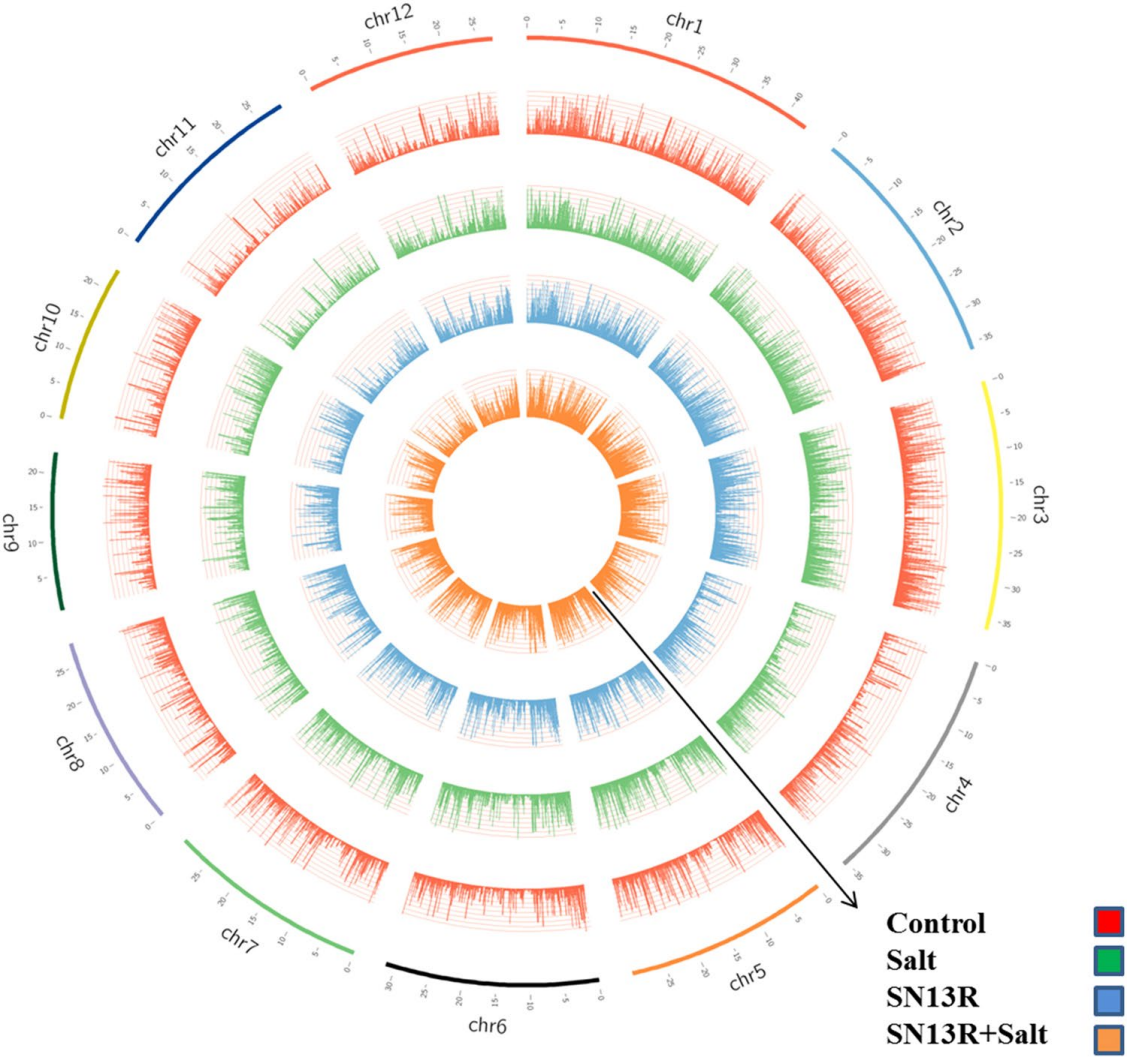
Circos shows the histogram of FPKM of genes mapped onto 12 rice chromosomes.

A total of 81,842,735 raw reads were generated through Illumina sequencing from the four libraries namely, control, SN13 and salt-treated rice seedlings with and without SN13 inoculation with an average of 20,460,684 reads per sample which remained unaltered after stringent quality check. Details of the Illumina sequencing raw data analysed through NGSQC ToolKit are listed in Table S3 and Tophat alignment summary is listed in Table S4. An average of about 16616299 (81.2%) reads mapped uniquely to the rice genome for each sample indicating good coverage of the rice genome. A total of 26401 (~84.7%) genes were found to be common in all the four libraries (Fig. 2a). Salt + SN13 sample (682) has the highest number of genes followed by salt (640) and control (367) treatments. The least number of genes (219) were identified in SN13 sample. Presence of such a high number of unique genes in salt + SN13 library as compared to other libraries indicates that bacterial inoculation extensively alters gene expression in rice seedlings under salt stress. A total of 2090 significant DEGs with log 2-fold change in the range of ≥+ 2.0 to ≤− 2.0 and P < 0.05 were identified from the comparative analysis of the three data sets (Control vs Salt, Control vs SN13, Control vs Salt + SN13). All genes from the four samples with expression patterns in the range of FPKM >1 and FPKM <300 were evenly mapped onto 12 rice chromosomes using circos plot (Fig. 3). On removal of duplicates 1523 DEGs were finally obtained out of which, 395 were up-regulated (≥ + 2.0-fold) in salt, 334 in SN13 and 755 in salt + SN13 treatments as compared to control (Fig. 2b). However, as compared to the up-regulated genes, less number of genes were found to be down-regulated in salt (155), SN13 (192), and salt + SN13 (259) treatments (Fig. 2c). However, overlaps of 27 DEGs were observed between transcriptional response to SN13 and salt, and of 113 between salt and salt + SN13 treatments as compared to control. While a significant overlap of 315 between SN13 and salt + SN13 transcriptional response was observed suggesting activation of a common regulatory pathway by SN13 in presence or absence of salt stress. The high number of altered expression genes obtained under salt + SN13 might be due to colonization of *B. amyloliquefaciens* and it might be a key element for induced systemic tolerance. The list of all DEGs along with their gene description and fold change values under all three conditions analyzed is given in the Table S5.

### Gene ontology, metabolic and regulatory pathway analysis of identified DEGs

Gene Ontology (GO) was used to assign putative functions to all identified DEGS into three principal categories, namely cellular component (1357), molecular function (1513), and biological process (2709) (Fig. S2a–c). An analysis of the GO terms suggested that ‘cellular component’ (15%), ‘membrane’ (14%), and ‘cell’ (11%) were the most dominant terms in the cellular component. While ‘molecular function’ (18%), ‘binding’ (15%) and ‘catalytic activity’ (14%) were the most abundant terms in the molecular function category. Similar observations were also reported in a recent study where a higher percentage of both binding and catalytic activity categories were observed for DEGs expressed in *Bacillus pumilus* treated *Radix* plants subjected to *Kosakonia sacchari* infection^28^. The enrichment analysis of DEGs identified several statistically significant GO terms for biological process category. The most highly represented subcategories included ‘metabolic process’ (15%), ‘cellular process’ (12%), ‘biological process’ (11%), ‘response to stress’ (8%), and ‘response to abiotic stress’ (5%). Moreover, only a few genes (~1% each) were categorized under ‘cell growth’, ‘cell death’, ‘ribosome’, ‘translation’, and ‘photosynthesis’. Several stress responses related biological process GO terms were significantly enriched in the up-regulated genes under salt and salt + SN13 treatments, such as ‘response to stress’, ‘response to abiotic stimulus’, ‘response to biotic stimulus’, and ‘response to extracellular stimulus’. These observations were in accordance to a previous study in *Arabidopsis* under treatment of *Burkholderia phytofirmans*, a PGPR, where genes falling under abovementioned GO terms were found to be upregulated^29^.

The DEGs were also examined against the KEGG database to detect biological pathways active in the rice roots in response to salt and SN13 treatments. Out of 1523 DEGs, only 297 were assigned to KEGG pathways under all treatments analyzed. Out of 297 DEGs, 89 were assigned to the metabolic pathways that comprised the largest category in all conditions as classified by KEGG (Table S6). The KEGG metabolic pathways largely constituted the biosynthesis of secondary metabolite; carbohydrate, lipid, amino acid, and nucleic acid metabolism; and energy metabolism in all three treatments. Interestingly KEGG pathway showed a high number of microbial metabolism in a diverse environment (ko01120) and also the presence of NOD-like receptor signaling pathway (ko04621) that indicates the involvement of SN13 in plant signaling under salt stress. Another study also described the role of the NOD-like receptor in microbial recognition^30^. Several other transcriptome studies also reported the higher number of microbial metabolism in the diverse environment under abiotic stress in the absence or presence of microbial association^31^. Flavonoid biosynthesis genes (ko00941) were also represented among other secondary metabolites biosynthesis genes confirming the role of flavonoids in root-rhizosphere signaling as reported earlier^32^.

**Figure 4:**
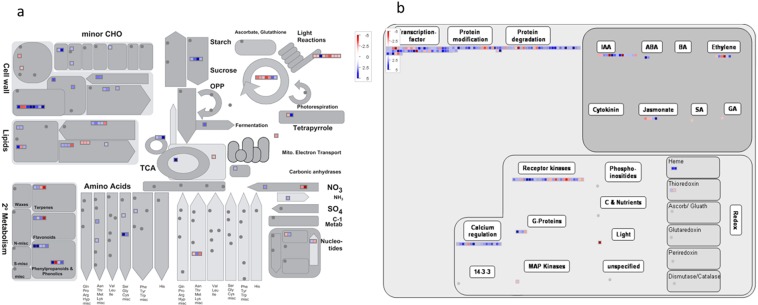
MapMan visualization for the observed differential expression patterns of transcripts involved in metabolic- (**a**) and regulatory pathways (**b**) in salt + SN13 conditions. In the display, each BIN or sub BIN is represented as a block where each transcript is displayed as a square. Red colors indicate down-regulation while blue colors showing up-regulation.

Since there was a significant overrepresentation of DEGs involved in signalling pathway including kinases and transcription factors (TFs) in response to all three conditions analysed, custom MAPMAN images were generated for the DEGs (p < 0.05, ≥ ± 2.0 fold) involved in metabolic pathways and regulatory functions (Fig. 4a,b and Fig. S3a–d). Interestingly higher number of PS II activity genes and other photosynthetic genes were found in SN13 treated plants under both stressed and unstressed condition. It is in accordance to the study of Cohen *et al*.^33^ that reported enhanced photosynthesis in drought-stressed *Arabidopsis* due to increase in chl a/chl b content or PS II activity in response to PGPR. Enhanced photosynthetic performance in *Arabidopsis* after inoculation of *Burkholderia phytofirmans* has also been reported^34^. While the exact mechanisms remain unclear, but higher number of photosynthetic genes and increased levels of chlorophyll content in salt + SN13 sample suggest that apart from bacterial ACD activity, elevated phosphorous and potassium uptake might play a role in this process which is in accordance to Dimkpa *et al*.^35^. Genes playing role in lipid, cell wall, phenolics, flavonoids and NO_3_^-^ metabolism were also represented in higher number in salt + SN13 sample than salt and SN13 samples individually which were in accordance to previous studies^36^. Such physiological changes are also typical indicators for induced systemic resistance (ISR)^35^. Remarkably genes encoding TFs, protein modification and degradation, receptor kinases and calcium regulating pathways were also found to be enriched in salt + SN13 samples as compared to salt and SN13 samples. Some receptor kinases like brassinosteroid insensitive 1 (BRI1, LOC_Os11g31530), S-locus receptor kinase (SRK, LOC_Os04g42740), lectin receptor protein kinases (LecRKs, LOC_Os11g03880) and two uncharacterized receptor protein kinases (LOC_Os02g57700 and LOC_Os03g36650) were also found to be unique DEGs in salt + SN13 sample indicating their possible crucial role(s) during beneficial plant-PGPR interaction. Higher expression of these genes was also reported under *Azospirillum-*rice cooperation^37^. Intriguingly genes regulating auxin, abscisic acid (ABA) and ethylene were also found to be enriched in salt + SN13 sample and are speculated to play a major role in plant growth and development. This could possibly be due to the fact that inoculation of plants with PGPR capable to produce such hormones could increase plant biomass and yield and stress conditions^6,33^.

### Genes regulated by salt stress in rice

Out of 64% genes that were uniquely expressed under salt stress only 52% genes could be functionally annotated. Among all functionally annotated genes TFs such as *AP2/ERF*, *MYB*, *bHLH*, *bZIP*, Zn-finger, and *G2*-like TF were highly represented. Several transcriptome studies have also revealed that TFs represent a broad category among regulatory genes under various abiotic stresses^37,38^. Ubiquitin/proteasomal protein degradation genes as well as secondary metabolites and stress-related genes were also found to be abundant under salt stress which is in accordance to a previous study on waterlogging stress in *Brassica napus*^39^.

### Genes regulated by SN13 inoculation in rice

Out of the total, 526 genes expressed in SN13 treated rice seedlings only 130 (25%) genes were found to be unique. However, functions of only 52% (68) of them were known of which photosynthetic genes were higher in percentage (29%) followed by protein modification and degradation (8%), TFs (5%), hormone metabolism (4%), amino acid metabolism (3%) and lipid metabolism (3%). An enhanced photosynthetic activity in *Arabidopsis* upon *Burkholderia phytofirmans* inoculation as well as an extensive alteration in expression patterns of genes involved in various metabolic pathways were observed in *Azospirillum brasilense* inoculated *Arabidopsis*^29,38^. Fascinatingly, no stress-responsive genes expressed in SN13 inoculated rice roots suggesting that SN13 is attributing favorable growth conditions resulting in overall better plant performance in agreement to several previous reports on PGPR attributes^29,36^.

### Genes regulated by salt and SN13 inoculation in rice

The highest number of genes was found to be regulated by the combination of salt stress and SN13 inoculation in rice seedlings as compared to either salt stress or SN13 inoculation. A total of 528 genes (52%) were found to be uniquely expressed in SN13 inoculated salt-stressed rice roots out of which only 55% genes are functionally known. Functions of the remaining 236 genes were not previously known suggesting them to be novel and hence could possibly open up newer research prospects for characterizing PGPR-responsive attributes. Genes regulating photosynthetic activity and TFs were found to be enriched as compared to salt treated samples. Spaepen *et al*.^38^ also reported enhancement of these traits in *Azospirillum brasilense* inoculated *Arabidopsis*. SN13 inoculation also led to a higher expression of auxin-responsive genes as well as genes regulating ethylene, jasmonate, gibberellin, salicylic acid metabolism under salt stress. This observation is in accordance to Spaepen *et al*.^38^ who reported a significant number of gene-regulating hormones in *Arabidopsis* inoculated with *Azospirillum brasilense*. Interestingly one GRAS-TF was also found to be expressed. Since GRAS-TFs play critical roles in plant growth and development via gibberellin and mycorrhizal signaling^40^, it is hypothesized that these TFs might play important role in rice-SN13 interactions too.

### Commonly differentially expressed genes under all conditions

Among all four libraries, a total of 511 genes were found to be common in at least any two libraries (Table S7). Heatmap was generated for different clusters of 511 genes using PageMan software (Fig. S4). As compared to salt stress, the stress-responsive genes namely, dehydrin (*DHN*) [LOC_Os11g26750, LOC_Os11g26780, LOC_Os11g26790], late embryogenesis abundant (*LEA*) [LOC_Os05g46480, LOC_Os08g23870] and universal stress protein (*USP*) [LOC_Os01g19820, LOC_Os01g2780, LOC_Os05g07810] were found to be highly induced (more than 4-fold) in SN13 inoculated rice roots under salt stress. A higher expression of these osmoprotectant genes in inoculated seedlings under stress condition indicates their crucial role in the biosynthesis of osmolytes and osmotic adjustment in response to SN13 which is in accordance to several previous reports^4,6,20^. In another study, *B. amyloliquefaciens* inoculation in rice under various abiotic stress and phytohormone treatments also showed the role of osmoprotectant genes in stress mitigation^20^. Recently, *Trichoderma harzianum*, a rhizosphere occupant was reported to play a vital role in stress alleviation in rice owing to the up-regulation of stress-responsive genes including *dehydrin*^41^. Likewise, an increased expression of *LEA* was observed upon *B. subtilis* treatment in *Brachypodium* under drought stress^42^ and upon *Pseudomonas putida* inoculation in chickpea^6^. Among various differentially expressed TFs, *bZIP* (LOC_Os06g41770) and *NAM* (LOC_Os05g10620, LOC_Os11g03370, LOC_Os12g03050) were up-regulated in SN13 inoculated seedlings under both stressed and non-stressed conditions. Gene expression profiling studies, also confirmed the PGPR-induced expression of *NAM* in chickpea and *Arabidopsis*, respectively^6,43^. Genes encoding GRAM domain containing protein (LOC_Os03g08860, LOC_Os12g29400) and non-symbiotic haemoglobin (LOC_Os03g12510, LOC_Os03g13140) were also found to be up-regulated in SN13 inoculated seedlings as compared to control. Bhattacharjee *et al*.^44^ also reported increased expression of GRAM domain containing protein under abiotic stresses while Bai *et al*.^45^ suggested the role of non-symbiotic haemoglobin in abiotic stress tolerance. The expression of gene encoding HKT1, which regulate Na^+^ import in plant root was also found to be downregulated in SN13 and salt + SN13 treated seedlings. According to Yang *et al*.^46^, VOCs secreted by *Bacillus*, downregulate HKT1 expression in roots for maintaining lower Na^+^ levels in the plant. Genes altering root system architecture (RSA) were also changed in inoculated condition under both stressed and unstressed conditions. The upregulation of Low phosphate root 1 (*LPR1*) gene (LOC_Os01g03530) indicates the modulation in the root system. This change in RSA might be due to the production of phytohormones, VOCs and other signalling molecules by SN13 that may lead to enhanced lateral root branching and development of root hairs^5,36,47^. Interestingly gene encoding for subtilisin-homolog (LOC_Os01g58240), a serine protease, was up-regulated in both SN13 and salt + SN13 conditions. An increased expression of subtilisin-like protease in *Bacillus subtilis* treated tobacco plants was also reported^48^. Similarly, increased expression of six nodulin genes was also observed under most of the stress conditions including salt stress. Interestingly several transporters such as aluminum-activated malate transporter (LOC_Os02g45160, LOC_Os04g47930), metal cation transporter (LOC_Os03g46470, LOC_Os05g39540), amino acid (LOC_Os08g03350, LOC_Os12g09300) and peptide transporters (LOC_Os10g02340) were found to be up-regulated (more than 3-fold) under SN13 and salt + SN13 conditions. The up-regulation of subtilisin, nodulins and transporter genes upon SN13 inoculation indicate the development of beneficial plant-microbe interactions as well as transport of useful compounds like amino acids, sugars, oligopeptides and polyamines through the root-rhizosphere interface from one organism to another indicating its role in salt stress alleviation in rice. A similar observation was also earlier reported during ectomycorrhiza and plant interaction^49^.

### Validation of DEGs through qRT-PCR

**Figure 5:**
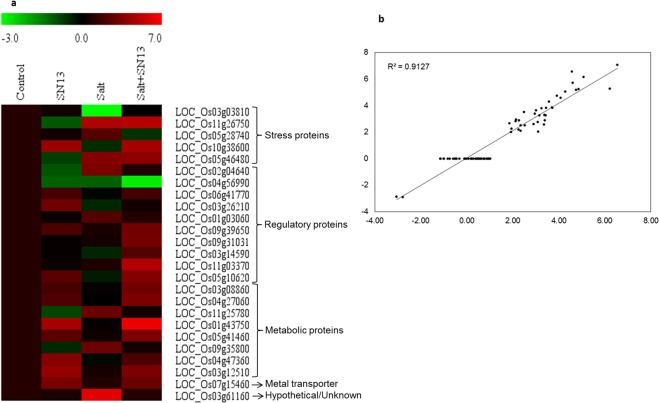
Differential expression of 25 genes through qRT-PCR analysis in rice exposed to salt stress in the presence or absence of SN13. The heat-map has been generated based on the fold-change values in the treated sample when compared with unstressed control sample. The colour scale for fold-change values is shown at the top (**a**). Scatters plot with correlation value of 0.91 between RNA-seq and qRT-PCR data (**b**).

To validate the RNA-seq value of differential gene expression, qRT-PCR was performed for 25 randomly selected DEGs representing different functional categories such as metabolism, regulation, stress-response, transporters and proteins with unknown functions (Fig. 5a). These genes were selected on the basis of their status of differential expression under one or more experimental condition(s) in rice roots from RNA-Seq data. Expression profiling through qRT-PCR revealed differential expression patterns for all 25 genes under all conditions. RNA-seq and qRT-PCR data showed a correlation value of 0.91 (Fig. 5b) for a total of 150 data points (average fold change in transcript levels of 25 genes under SN13, salt and salt + SN13 treatments in rice roots), indicated a very good agreement between real-time PCR analysis with respect to the transcriptomic data, further confirming the potential of high-throughput NGS technologies for gene expression quantification.

### Expression of OsNAM and OsGRAM gene in yeast

**Figure 6:**
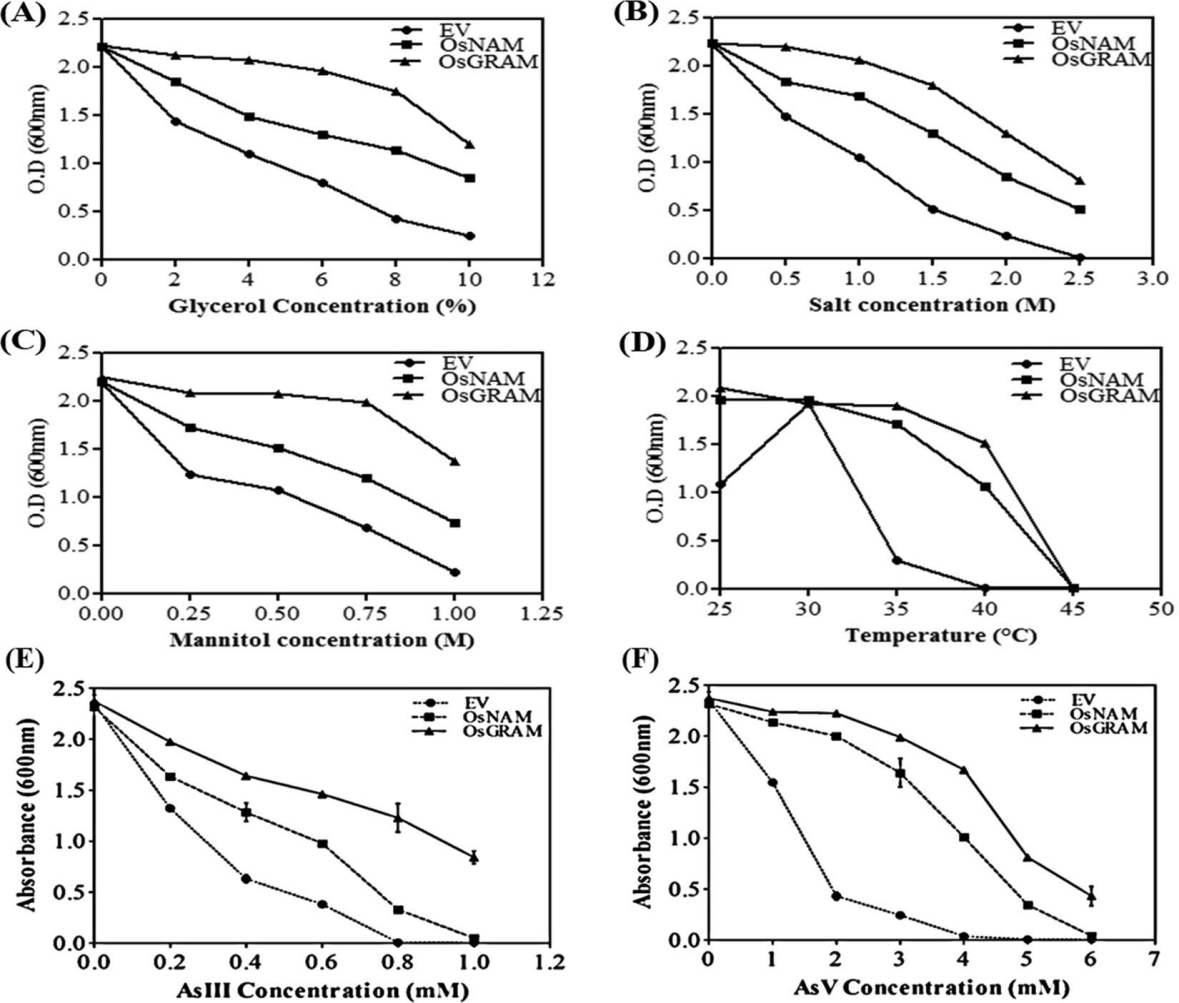
Expression of *OsNAM* and *OsGRAM* increased tolerance in *S. cerevisiae* (INVSc1). S. cerevisiae (INVSc1) cells transformed with empty vector (pYES2), *OsGRAM* and *OsNAM* were cultured in YPD medium with 2% galactose containing (**a**) Glycerol, (**b**) NaCl, (**c**) Mannitol, (**d**) high temperature, (**e**) Arsenite and (**f**) Arsenate. Growth was monitored spectrophotometrically at 600 nm.

A gene encoding *NAM* and *GRAM* with ~5-fold and ~3-fold induction respectively in transcriptome sequencing of salt + SN13 rice root sample was chosen for functional validation. For functional analysis, the full-length cDNAs of *OsNAM* (1080 bp) and *OsGRAM* (795 bp) were cloned in yeast pYES2 vector and transformed in *Saccharomyces cerevisiae* strain INVSc1 under the control of the Gal-inducible GAL1 promoter. Our results indicated that yeast cells expressing *OsNAM* and *OsGRAM* showed enhanced tolerance under all three osmotic stresses i.e. glycerol, mannitol and NaCl, as well as better able to survive at higher temperatures i.e. 35 °C and 40 °C though growth was lesser at 40 °C and cell death occurred at 45 °C as compared to strain carrying EV (Fig. 6a–d). Interestingly both the genes also showed better tolerance to different concentration of arsenite (AsIII) and arsenate (AsV) in transformed yeast in comparison of EV transformed yeast (Fig. 6e,f). Interestingly, yeast cells expressing *OsGRAM* showed significantly more tolerance (~2-fold) to all osmotic stresses as compared to cells expressing *OsNAM* at all concentrations, and also performed better at high-temperature stress as compared to *OsNAM* overexpressing cells indicating the important role of *OsGRAM* in providing stress tolerance to yeast. *OsNAM* belong to NAC (NAM, ATAF, CUC) TFs superfamily, known to be associated with various biological processes along with biotic and abiotic stress tolerance^50–52^ but their role in stress alleviation in plants under the influence of any PGPR is yet not explored. Similarly, the role of GRAM family members in PGPR-mediated stress amelioration in plants is not reported, however, a few earlier studies reported that, *GRAM* domain containing genes are important for ABA response, perception and regulation of environmental and hormonal signalling under both biotic and abiotic stress conditions^53–55^. Many soil microorganisms including *Bacillus* regulate the overall hormonal balance in plants and their responses to various stresses via production and modulation of endogenous levels of phytohormones^20,56^. Considering these facts along with the complementation studies in yeast indicated the possibility of improved abiotic stress tolerance by *OsNAM* and *OsGRAM* genes in plants under the influence of SN13 either through modulation of phytohormones or by regulating other stress-responsive genes/small RNAs which need detailed investigations in near future. This is thus the first report on the functional characterization of both genes in improving abiotic stress tolerance in yeast.

### Conclusion and future perspectives

**Figure 7:**
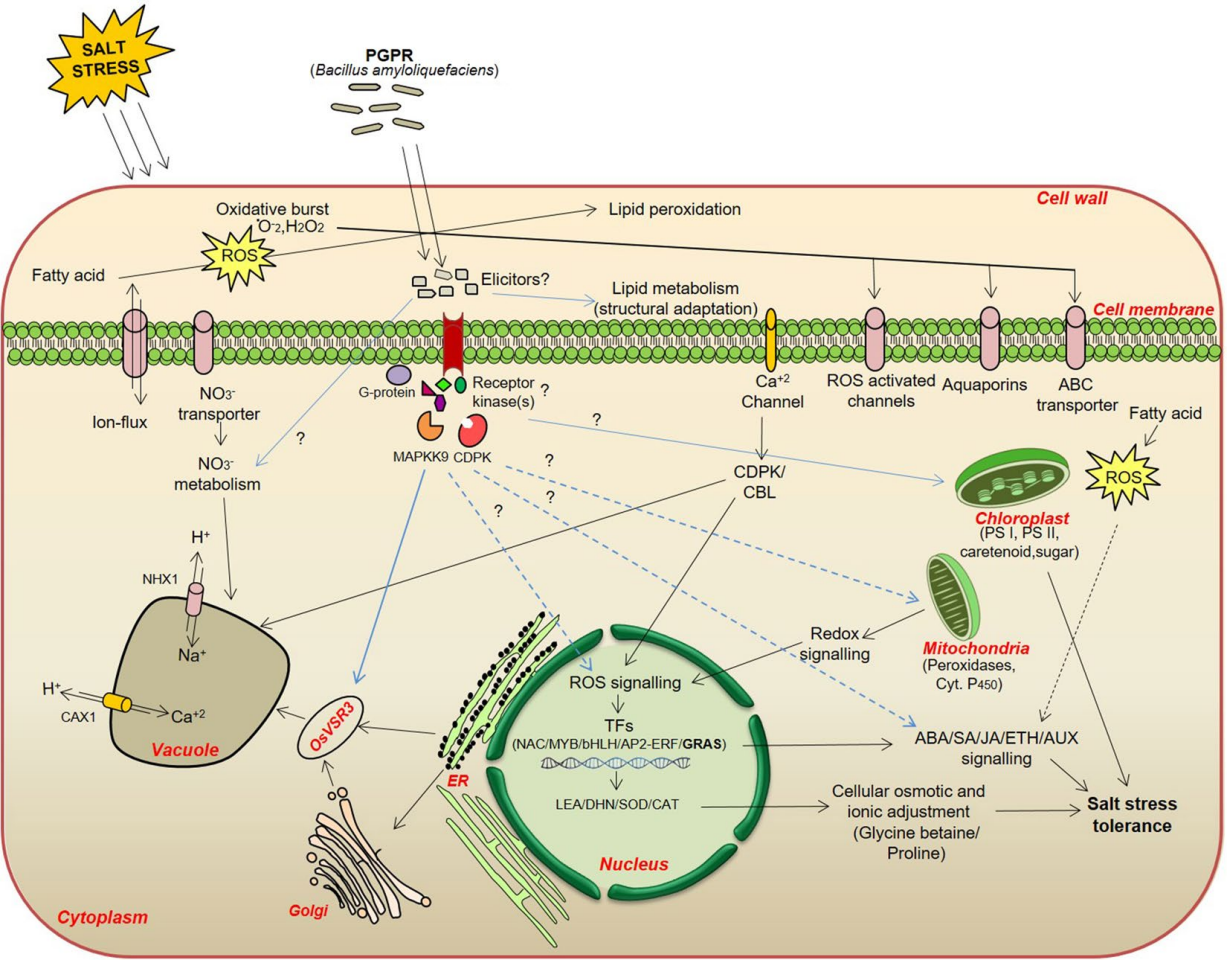
A model depicting SN13-mediated salt stress regulation in rice seedlings. Solid and dashed blue lines indicate stress regulation based on uniquely found genes in SN13 treated seedlings under salt stress while solid and dashed black arrows indicate stress regulation based on commonly found genes in both salt and salt + SN13 seedlings according to well-known concepts reported earlier. Dashed arrows indicate the existence of not well-characterized signalling pathways. The SN13-mediated salt stress regulation shown here is only a fraction of stress signalling occurring in plants and different other signalling and crosstalk points are yet to be discovered and characterized.

**Figure 8:**
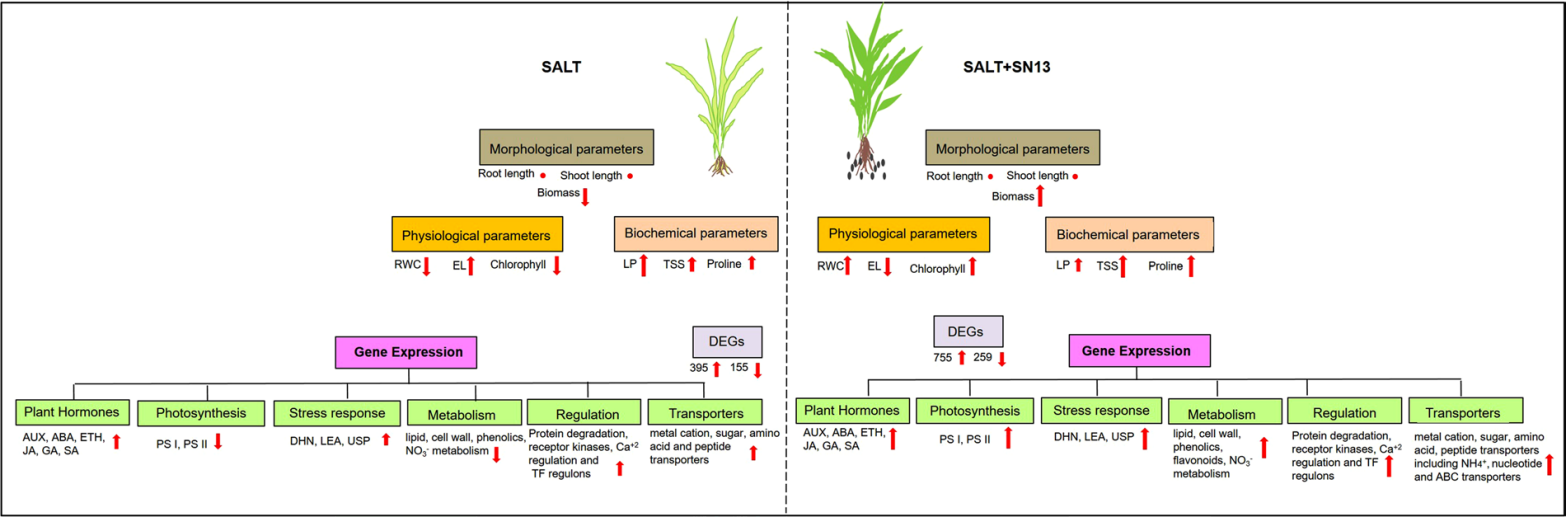
A figure illustration summarizing the overall findings from this study. Upward arrows indicate enhanced while down-ward arrows indicate decreased morphological, physiological and molecular parameters. Dot symbol represents no significant changes. Size of the arrows indicates comparative quantification between parameters.

In the present study, a detailed molecular mechanism of *B. amyloliquefaciens* mediated “plant-microbe-stress” interaction was established. Water status, membrane integrity, accumulation of osmolytes, photosynthetic activity and gene expression in rice was found to be significantly improved in the presence of SN13 under salt stress condition. The comparative transcriptome study revealed that the highest number of genes was regulated in the presence of SN13 under salt stress. Exclusively this is the first report on the analysis of DEGs in rice upon PGPR inoculation under salt stress that includes genes reported previously as well as unknown genes. Extensive alteration of metabolic and regulatory pathway genes in salt + SN13 library indicates its substantial role in influencing the overall homeostasis of rice seedlings under salt stress. Further, the activation of these genes suggests their role most likely in the regulation of salt stress adaptation in this crop upon SN13 inoculation. The presence of large number of DEGs with no known functions also explains the uniqueness of plant-microbe interaction under salt stress. Based on unique DEGs found in salt + SN13 sample along with previous literature and established concepts, a functional hypothesis for the mechanism of PGPR-mediated salt stress tolerance in rice has also been elaborated (Fig. 7). It has been hypothesized that rhizobacteria-mediated signalling pathway initiates with release of certain bacterial elicitor(s) and their perception by plasma membrane receptor kinases or by bacterial elicitors-mediated direct influence on cellular metabolism such as lipid metabolism, nitrate metabolism etc. Further, receptor kinases trigger the downstream signalling cascade that include phosphorylation and dephosphorylation mediated by several protein kinases and phosphatases. These protein kinases such as Ca^2+^-dependent (CDPK) and mitogen activated (MAPKs) protein kinases transduce the signals to different cell organelles including nucleus, mitochondria, and chloroplast that could be either directly involved in protection of cellular machinery or regulation of gene expression under stress. Our study thus provides novel insights into the plant response at the molecular level on *B. amyloliquefaciens* inoculation suggesting it as a potential microbe to be used as bioinoculants as well as highlights its applicability in salt stress amelioration at field level. Overall findings from this study have also been summarized in Fig. 8. In conclusion, this work led to the identification of several PGPR-responsive transcripts in rice under salt stress and functional characterization of two *OsNAM* and *OsGRAM* genes is useful in expanding our understanding of the molecular basis plant-PGPR interaction in rice under salt stress. However, extensive studies still need to be taken up to unravel the molecular mechanisms of plant-SN13 interaction and stress tolerance. This progress ought to bring a huge wealth of information on novel PGPR-responsive genes related to abiotic stress tolerance to the research community which will not only be very helpful in understanding basic plant biology but also in developing improved crop varieties.

## Supplementary information

Supplementary Information

Table S5

Table S6

Table S7
