# Supplementary material for: Transcriptional alterations reveal Bacillus amyloliquefaciens-rice cooperation under salt stress
Source: Sci Rep. 2019 Aug 15;9:11912. doi: 10.1038/s41598-019-48309-8 (PMC6695486; doi:10.1038/s41598-019-48309-8)
Supplement: Supplementary file 1 — Supplementary Information [file 41598_2019_48309_MOESM1_ESM.pdf]

Transcriptional alterations reveal *Bacillus amyloliquefaciens*-rice cooperation under salt stress

Puneet Singh Chauhan[1,2#], Charu Lata[1,2#], Shalini Tiwari[1], Abhishek Singh Chauhan[1,2], Shashank Kumar Mishra[1,2], Lalit Agrawal[1], Debasis Chakrabarty[1,2], Chandra Shekhar Nautiyal[1]*

[1]*CSIR-National Botanical Research Institute, Rana Pratap Marg, Lucknow 226001, India*

[2]*Academy of Scientific and Innovative Research (AcSIR), 2 Rafi Marg, New Delhi 110001, India*

[#]Authors contributed equally as first author

Correspondence may be addressed to

Dr. Chandra Shekhar Nautiyal

Division of Plant Microbe Interactions

CSIR-National Botanical Research Institute

Rana Pratap Marg, Lucknow 226 001, India

E-mail: csnnbri@yahoo.com

Phone: +91-522-2205848

Fax: +91-522-2205839

Fig. S1: Phenotype of rice seedlings exposed to salt stress in the presence and absence of SN13.

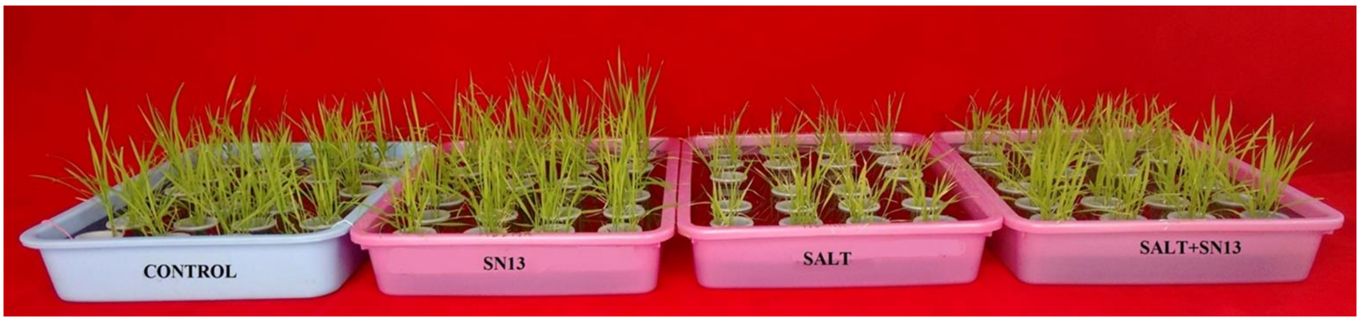

Fig. S2: Blast2GO term assignment of the differential expressed genes in different categories of biological process (a), cellular function (b) and molecular component (c)

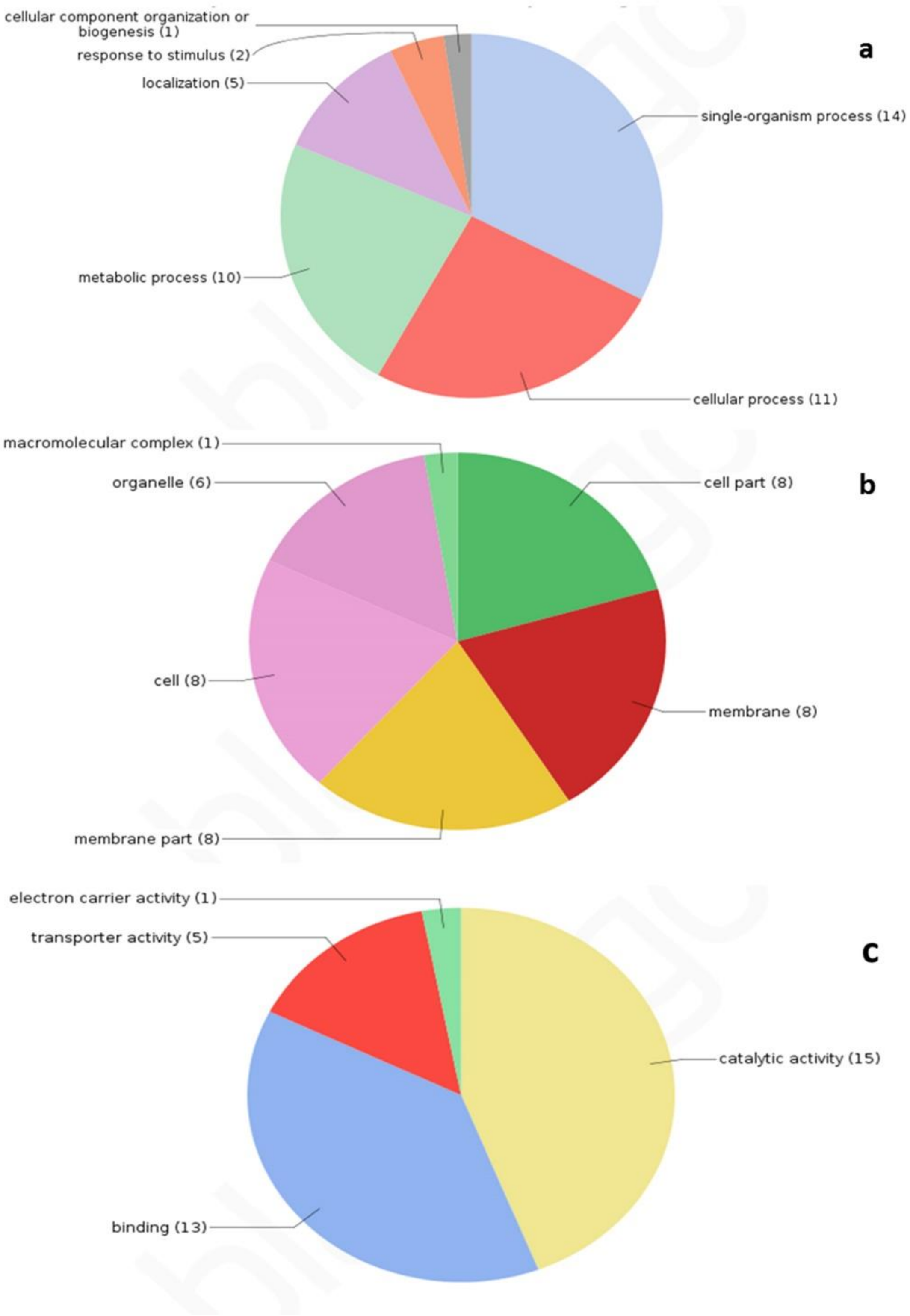

Fig. S3: MapMan visualization for observed differential expression patterns of transcripts involved in metabolic and regulatory pathways in salt (a,b) and SN13 (c,d) treatments.

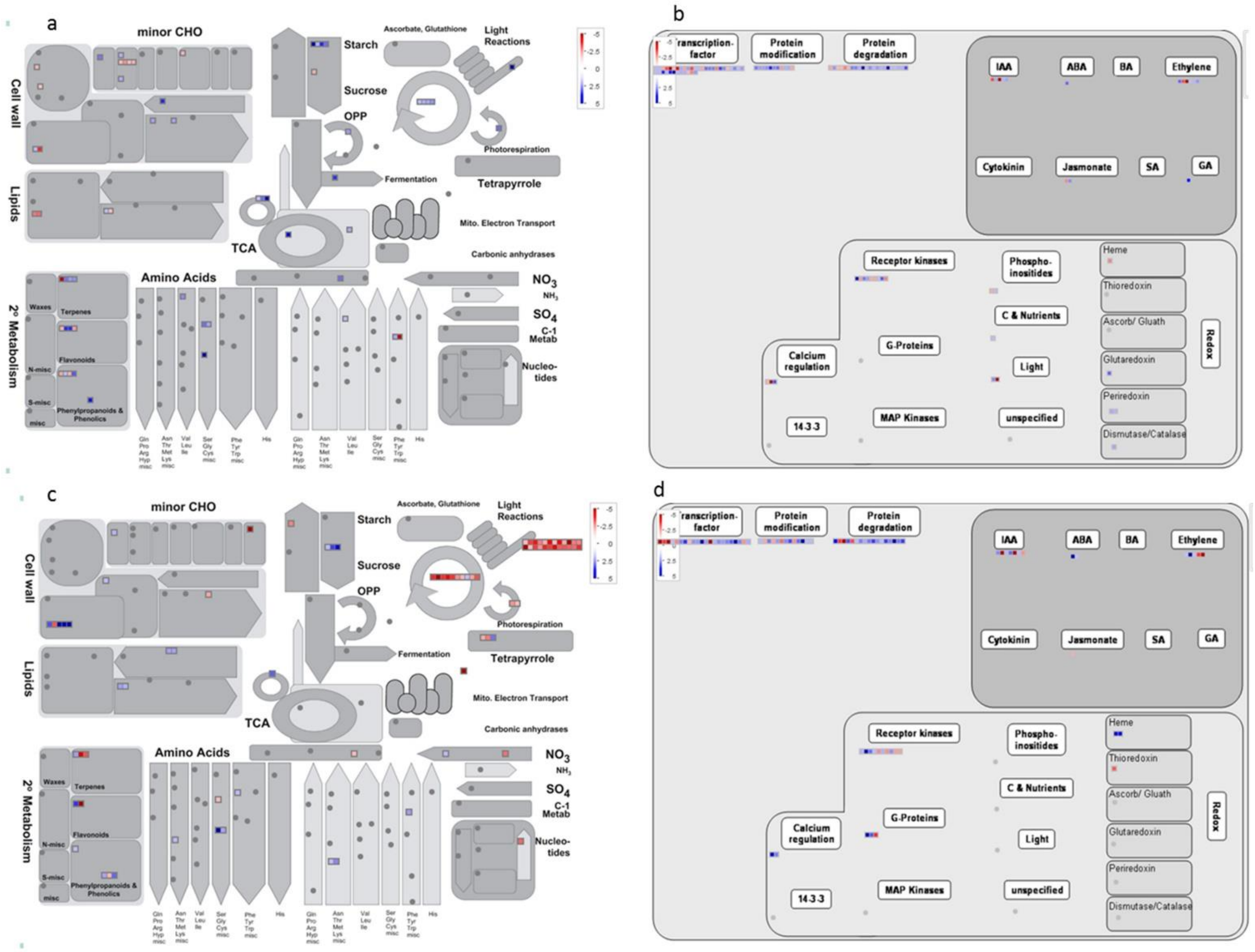

Fig. S4: A heat map showing under and over represented functional classifications among genes which were differentially regulated. This analysis was performed using the PageMan software package. Red boxes indicate that genes in a category were generally down-regulated while blue boxes indicate that genes in a category were generally up-regulated in the corresponding set of samples relative to controls.

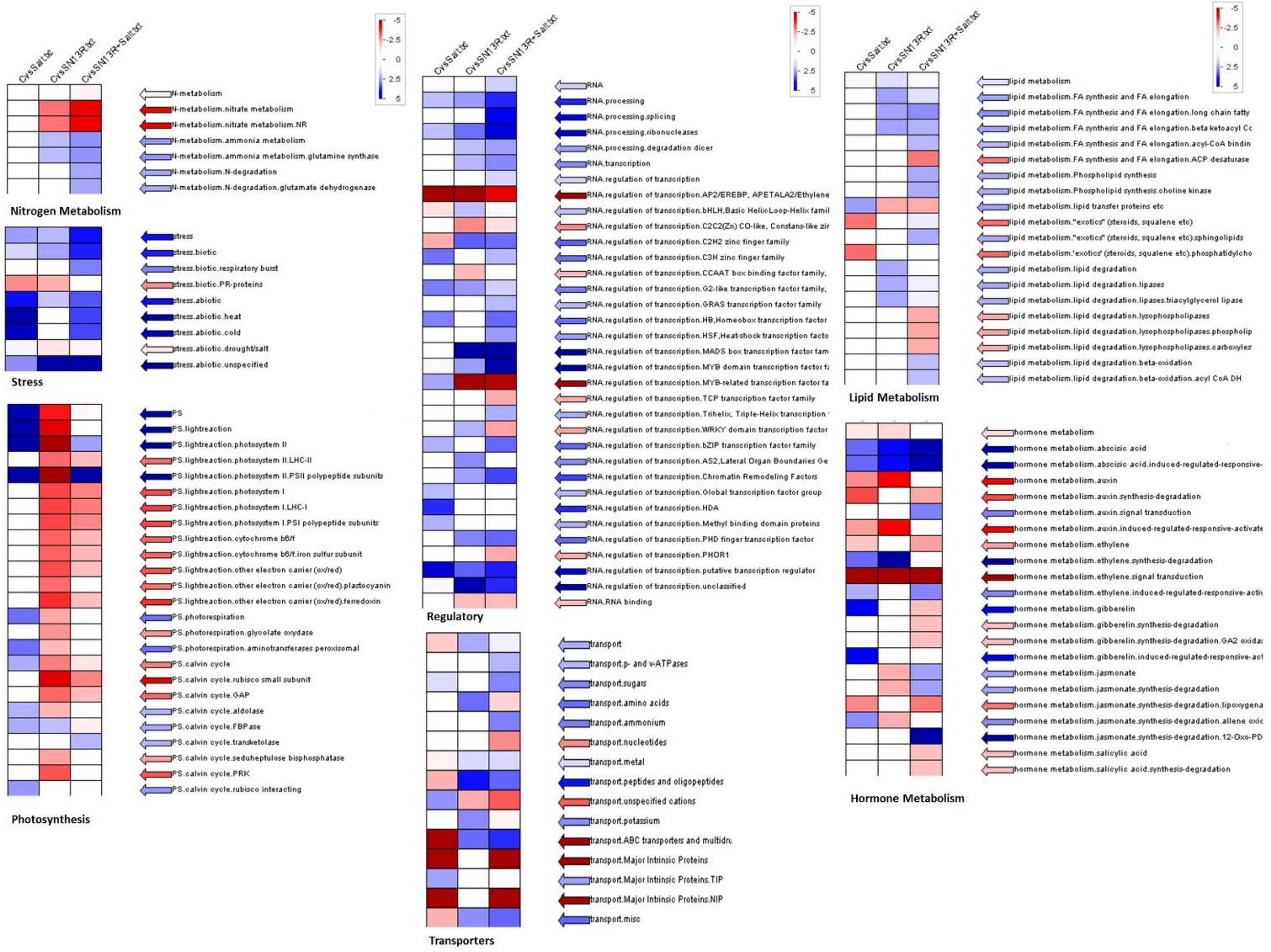

Table S1: List of primers used in the study

Gene(s)	Description	Accession no.*	Primer Sequence	Amplicon Length
DEF	qRT-PCR	LOC_Os03g03810	F-5' CTTCACGCAAGGTGTTCTC 3'<br>R-5' CTTGAACCGGTGGCTCT 3'	125
DHN	,,	LOC_Os11g26750	F-5' GCAACAAGGGCAACAAC 3'<br>R-5' GGTGGCGATCTTGGTATC 3'	98
USP	,,	LOC_Os05g28740	F-5' GAGCCATGGTTACGGTTTC 3'<br>R-5' CCTCTTGACGACGACGA 3'	97
GSTU6	,,	LOC_Os10g38600	F-5' GCCTACGTCACCAGAGT 3'<br>R-5' AGGAGGTCGCTCTTGTT 3'	92
LEA	,,	LOC_Os05g46480	F-5' TTCCTCGGCGAGAAGAC 3'<br>R-5' GGCAGAGTCCTTGGTGTA 3'	105
Myb	,,	LOC_Os02g04640	F-5' GTCTGAACAGCTCGGAATTG 3'<br>R-5' GCAAGATGACACTGGAAGAG 3'	106
Myb	,,	LOC_Os04g56990	F-5' CAACAGCATCAGGACAGAAT 3'<br>R-5' CTTGAGGTGGAAACGAGTG 3'	97
bZIP	,,	LOC_Os06g41770	F- 5' CGACGAGTCCGCCATATTTA 3'<br>R- 5' GTCGGCGAAGAACTCTACAT 3'	103
HLH	,,	LOC_Os03g26210	F- 5' GAGAGGGAGAAGCTTAAACG 3'<br>R- 5' GGGTGGTGTCAGTTAGTATG 3'	115
Splicing Factor	,,	LOC_Os01g03060	F- 5' CTCAGACTCAAGCGATGATAC 3'<br>R-5' GATGCTTTCTCCTGTGTGAG 3'	95
Protein kinase	,,	LOC_Os09g39650	F- 5' ACGATGATGTTCTTGCTGGTGTT 3'<br>R-5'TGACATGCTCCTTGAACCAATG 3'	83

UBQ	,,	LOC_Os09g31031	F- 5' TCACCACTGCCGCAAGAAG 3'<br>R-5' GCTATCGAAAGCGAGTTGATGAA 3'	81
Ca-EF hand	,,	LOC_Os03g14590	F- 5' CTGAGCAACATCGCTGAAAGG 3'<br>R-5' GTTCTCGGTGTGTGCGACAA 3'	60
NAM	,,	LOC_Os11g03370	F-5' CAGATGCGGAGGTAATCTTG 3'<br>R-5' CATGGCTCGCACTTGTT 3'	103
NAM	,,	LOC_Os05g10620	F-5' CACTTCAGCTCAGCTCCTACCAA 3'<br>R-5' AGTGGTGACCCCGATCAACTT 3'	79
Aldo/keto reductase	,,	LOC_Os04g27060	F-5' TTCCGCAAGAGCCTACCTAGATT 3'<br>R-5' CCATGGCATTAACCTTCTCGAA 3'	79
GRAM domain	,,	LOC_Os03g08860	F-5' CAGGTGGTTTCCGTTGATAA 3'<br>R-5' GAAGTGCTTCCTGCAGATT 3'	88
PB1	,,	LOC_Os11g25780	F-5' CTGTACCACCGTCAGACT 3'<br>R-5' GTTGTGATGCTGCTGCTG 3'	108
Cyt. P450	,,	LOC_Os01g43750	F-5' CCGTAACCTTCACTCGAAAG 3'<br>R-5' TGGGTCATGGTGTATGAGTA 3'	107
Cystein proteinase inhibitor	,,	LOC_Os05g41460	F- 5' AATAAGCTGTACGAGGCCAAGGT 3'<br>R- 5' TGAAATCCTGGAGCTGCTTGA 3'	67
UGE	,,	LOC_Os09g35800	F- 5'GGAGATTGCACTGAGGTTT 3'<br>R- 5' CCTGCACATGTCCTCTATTC 3'	90
POP9	,,	LOC_Os04g47360	F- 5' GCTTGTGCAGTCCATGGTGATA 3'<br>R-5' CAAGCTCCTCATCTGCAAGACA 3'	78
NSHB	,,	LOC_Os03g12510	F- 5' CATTGGACTCCGCTTCTTCTTG 3'<br>R-5' AGTTGCGCAGGAACGAGAAC 3'	77
Metal transporter	,,	LOC_Os07g15460	F- 5' CTACTTCCTCAGCACAAAGC 3'<br>R-5' TTCCTGAACGTCAGGTAGAT 3'	138
Actin	,,	LOC_Os11g06390	F-5' CGGTGTGATGGTTGGTATG 3'<br>R-5' ATGCTCGATCGGGTACTT 3'	97
OsNAM	Full length cloning		F1-5' GCATGGTGGAGACTAGTACA 3'<br>R1-5' GCACTAGCTATTACGATGAGTC3'<br>F2-5' ATGGTGGAGACTAGTACATCC3'<br>R2-5' GCTATTACGATGAGTCCCATAG 3'	1080
OsGRAM	,,		F1-5' TCCCCGCCGTCGACATGCAC3'<br>R1-5' CTATGGACATCACTAAGCTCGTAG 3'<br>F2-5' ACATGCACCCGCCGGCCG3'<br>R2-5' CTAAGCTCGTAGATTCTGAGCCTCC3'	795
OsNAM_BamHI_F<br>OsNAM_XbaI_R	Yeast cloning	-	F-5'CGGGATCCCGATGGTGGAGACTAGTACATCC 3'<br>R-5' GCTCTAGAGCGCTATTACGATGAGTCCCATAG 3'	
OsGRAM_BamHI_F<br>OsGRAM_XbaI_R	.	-	F-5'CGGGATCCCGACATGCACCCGCCGGCCG3'<br>R-5'GCTCTAGAGCTAAGCTCGTAGATTCTGAGCCTCC3'	
M13	Sequencing	-	F-5'CGCCAGGGTTTTCCCAGTCACGAC3'<br>R-5'TCACACAGGAAACAGCTATGAC3'	-

*The Institute of Genomic Research (TIGR)-Rice Genome Annotation Project locus ID.

Table S2: Effect of NBRI-SN13 on the growth parameters of rice cultivars subjected to salt stress.

Treatments	Shoot length (cm.)	Root Length (cm.)	Fresh weight (mg.)	Dry weight (mg.)
Control	16.25±1.7 b	6.48±1.25 a	116.35±14.9 c	5.55±1.20 a
SN13	17.45±0.98 b	8.00±0.67 b	223.00±13.8 d	10.53±1.28 b
Salt	8.58±0.85 a	7.65±0.73 ab	57.23±5.0 a	5.73±0.59 a
Salt+SN13	9.25±0.47 a	6.43±0.72 a	75.98±9.7 b	7.28±1.40 a

Table S3: A NGSQCTOOL kit result for control, salt, SN13, salt+SN13 samples.

**File name**	**Control_1.fq**	**Control_2.fq**	**Control_1.fq_ filtered**	**Control_2.fq_ filtered**
Minimum read length	100	100	100	100
Maximum read length	100	100	100	100
Average read length	100	100	100	100
Total number of reads	16568439	16568439	16568439	16568439
Total number of reads with non-	24027	2287	24027	2287

ATGC bases				
Percentage of reads with non-ATGC bases	0.15%	0.01%	0.15%	0.01%
Total number of bases	1656843900	1656843900	1656843900	1656843900
Total number of HQ bases	1637763602	1633570766	1637763602	1633570766
Percentage of HQ bases	98.85%	98.60%	98.85%	98.60%
Total number of non-ATGC bases	24669	3760	24669	3760
Percentage of non-ATGC bases	0.00%	0.00%	0.00%	0.00%
**File name**	**Salt_1.fq**	**Salt_2.fq**	**Salt_1.fq_ filtered**	**Salt_2.fq_ filtered**
Minimum read length	100	100	100	100
Maximum read length	100	100	100	100
Average read length	100	100	100	100
Total number of reads	23387476	23387476	23387476	23387476
Total number of reads with non-ATGC bases	34117	3333	34117	3333
Percentage of reads with non-ATGC bases	0.15%	0.01%	0.15%	0.01%
Total number of bases	2338747600	2338747600	2338747600	2338747600
Total number of HQ bases	2312807360	2305168395	2312807360	2305168395
Percentage of HQ bases	98.89%	98.56%	98.89%	98.56%
Total number of non-ATGC bases	34958	5374	34958	5374
Percentage of non-ATGC bases	0.00%	0.00%	0.00%	0.00%
**File name**	**SN13_1.fq**	**SN13_2.fq**	**SN13_1.fq_ filtered**	**SN13_2.fq_ filtered**
Minimum read length	100	100	100	100
Maximum read length	100	100	100	100
Average read length	100	100	100	100
Total number of reads	17739411	17739411	17739411	17739411
Total number of reads with non-ATGC bases	26065	2505	26065	2505
Percentage of reads with non-ATGC bases	0.15%	0.01%	0.15%	0.01%
Total number of bases	1773941100	1773941100	1773941100	1773941100
Total number of HQ bases	1754884582	1749699923	1754884582	1749699923
Percentage of HQ bases	98.93%	98.63%	98.93%	98.63%
Total number of non-ATGC bases	26703	4184	26703	4184
Percentage of non-ATGC bases	0.00%	0.00%	0.00%	0.00%
**File name**	**SN13+Salt_1.fq**	**SN13+Salt_2.fq**	**SN13+Salt_1.fq_ filtered**	**SN13+Salt_2.fq_ filtered**
Minimum read length	100	100	100	100
Maximum read	100	100	100	100

length				
Average read length	100	100	100	100
Total number of reads	24147409	24147409	24147409	24147409
Total number of reads with non-ATGC bases	35741	3466	35741	3466
Percentage of reads with non-ATGC bases	0.15%	0.01%	0.15%	0.01%
Total number of bases	2414740900	2414740900	2414740900	2414740900
Total number of HQ bases	2388732530	2382207391	2388732530	2382207391
Percentage of HQ bases	98.92%	98.65%	98.92%	98.65%
Total number of non-ATGC bases	36664	5768	36664	5768
Percentage of non-ATGC bases	0.00%	0.00%	0.00%	0.00%

Table S4: TopHat Alignment Summary for control, salt, SN13, salt+SN13 samples.

**Samples**	**Left Reads**			**Right Reads**		
	HQ Reads	Aligned Reads	Unaligned Reads	HQ Reads	Aligned Reads	Unaligned Reads
Control	16568439	14176033 (85.6%)	2392406 (14.4%)	16568439	14145712 (85.4%)	2422727 (14.6%)
Salt	23387476	19811116 (84.7%)	3576360 (15.3%)	23387476	19761936 (84.5%)	3625540 (15.5%)
SN13	17739411	15616276 (88%)	2123135 (11.9%)	17739411	15584732 (87.9%)	2154679 (12.1%)
SN13+ Salt	24147409	20070035 (83.1%)	4077374 (16.9%)	24147409	20029887 (82.9%)	4117522 (17%)
